# Combined Treatment with Antiviral Therapy and Rituximab in Patients with Mixed Cryoglobulinemia: Review of the Literature and Report of a Case Using Direct Antiviral Agents-Based Antihepatitis C Virus Therapy

**DOI:** 10.1155/2015/816424

**Published:** 2015-03-01

**Authors:** Teresa Urraro, Laura Gragnani, Alessia Piluso, Alessio Fabbrizzi, Monica Monti, Elisa Fognani, Barbara Boldrini, Jessica Ranieri, Anna Linda Zignego

**Affiliations:** Center for Systemic Manifestations of Hepatitis Viruses (MASVE), Department of Experimental and Clinical Medicine, University of Florence, Lagro Brambilla 3, 50134 Florence, Italy

## Abstract

Mixed cryoglobulinemia (MC) is an autoimmune/B-cell lymphoproliferative disorder associated with Hepatitis C Virus (HCV) infection, manifesting as a systemic vasculitis. In the last decade, antiviral treatment (AT) with pegylated interferon (Peg-IFN) plus ribavirin (RBV) was considered the first therapeutic option for HCV-MC. In MC patients ineligible or not responsive to antivirals, the anti-CD20 monoclonal antibody rituximab (RTX) is effective. A combined AT plus RTX was also suggested. Since the introduction of direct acting antivirals (DAAs), few data were published about MC and no data about a combined schedule. Here, we report a complete remission of MC after a sustained virological response following a combined RTX/Peg-IFN+RBV+DAA (boceprevir) treatment and review the literature about the combined RTX/AT.

## 1. Introduction

Mixed cryoglobulinemia (MC) is both an autoimmune and B-cell lymphoproliferative disorder (LPD) characterized by immune complexes (cryoglobulins, CGs) that reversibly precipitate at low temperature [1].

In the large majority of cases, MC is triggered by Hepatitis C Virus (HCV) infection.

HCV is both a hepatotropic and lymphotropic virus and may lead not only to liver diseases, but also to some lymphoproliferative disorders ranging from the benign MC to malignant B-cell non-Hodgkin's lymphoma [2].

Small-to-medium sized vessel vasculitis is the pathological substrate of the MC-related clinical syndrome (MCS) [1].

Antiviral therapy (AT) has been indicated as first-line therapy in patients with mild-to-moderate HCV-related MCS. The use of IFN-*α* to treat MC was successfully proposed, even before HCV discovery, because of its antiproliferative and immunomodulatory effects [3]. However, the benefit was transient and relapses were very frequent after treatment discontinuation when viral eradication was not achieved [4–6].

Starting with the IFN monotherapy, the efficacy of AT progressively increased during two decades thanks to the use of Peg-IFN and RBV. The increased antiviral activity was associated with an increased clinical effectiveness in patients with MCS [2, 7, 8].

In severe cases and/or in patients intolerant or ineligible to AT, the usefulness and safety of anti-CD20 monoclonal antibody rituximab (RTX) have been clearly shown in several studies. RTX was shown to be highly effective in modifying the dynamics of B cells by deleting expanded clones and markedly improving MCS in most cases [9–11].

Peg-IFN plus RBV has been considered the standard AT for about a decade [12]. Recently, the direct acting antiviral drugs (DAAs), inhibitor of the nonstructural 3/4A HCV protease, boceprevir (BOC), and telaprevir, in combination with Peg-IFN and RBV, have consistently increased the likelihood of response in patients infected with HCV genotype 1 (Gt 1a or 1b) [13, 14]. Few data are still available for patients with HCV-related MCS treated with DAAs [15–18] and no clear information exists about the combination with RTX.

Here we describe, for the first time, a case of HCV-related MCS treated with the combination of RTX and BOC-based triple AT and review the available literature about the combination of RTX and anti-HCV therapy.

## 2. Case Report

In May, 2010, a 63-year-old woman with a 30-year history of HCV (Gt 1b) chronic infection and harboring compensated liver cirrhosis with normal liver function tests and no signs of portal hypertension was evaluated at the MASVE Center of the University of Florence. The main clinical and laboratory data are detailed in Table 1. The analysis of cryocrit was performed in the MASVE laboratory at every medical appointment. Briefly, the patient blood was kept at 37°–40°C to avoid CGs precipitation; immediately after centrifugation, a special graduated glass tube (Wintrobe tube) has been filled with 1.0 mL of serum, placed at 4°C, and examined after at least 7 days to assess the percentage of packed cryoglobulins.

The reduction of C4 complement component and the RF levels was performed as routine tests by the Centralized Diagnostic Laboratory of the Careggi Hospital, Florence, Italy, by immunoturbidimetric method and immunoenzymatic assay, respectively.

Since 2008, the patient showed a typical MCS [19] characterized by recurrent lower limb purpura, arthralgia/arthritis of hands and shoulders, and burning paresthesia with “bootie” distribution. She repeatedly scored positive for mixed type II (polyclonal IgG/monoclonal IgMk) cryoglobulins as well as rheumatoid factor (RF), increased inflammatory markers, and low C4. The patient previously failed two Peg-IFN*α*-2b+RBV treatments. Her interleukin-28b (IL28b) genotype (rs12979860) was T/T, a negative predictor of response to IFN-based therapy [20]. In June 2010, the patient underwent treatment with RTX (1 g twice monthly) which led to a complete MCS clinical response, with improvement in all baseline clinical manifestations [21]. After recurrence of symptoms in July 2011, the treatment was repeated. Clinical conditions consistently improved and in September 2011 the patient underwent AT. The BOC-based treatment was chosen on the basis of negative predictors of response [12] and higher efficacy of triple therapy compared to dual one.

After a 4-week lead-in phase with Peg-IFN*α*-2b (1.5 *μ*g/Kg/week, subcutaneously) plus RBV (800 mg/daily, orally), BOC was added (800 mg three times daily, orally) and triple therapy was continued for 44 weeks.

Viremia disappeared 1 week after BOC administration and remained negative throughout therapy and follow-up (sustained virological response).

**Figure 1:**
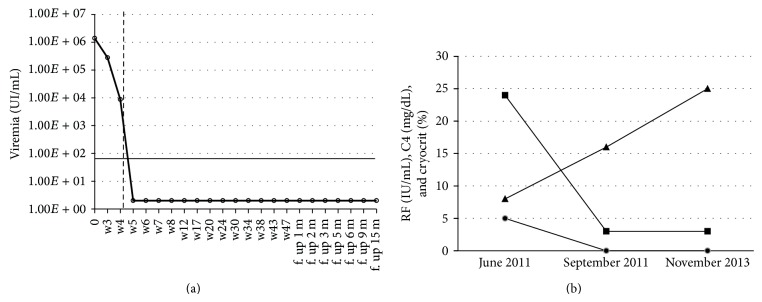
(a) Viral kinetics: a slight decrease in serum HCV RNA values was observed during the lead-in phase. Soon after the introduction of boceprevir (week 4), a drastic reduction in viremia was observed, with complete negativization at week 5. The vertical dashed line represents the introduction of boceprevir; the horizontal continuous line indicates the detection limit of the HCV RNA test (15 IU/mL). IU: international units; mL: milliliter; w: week; f. up: follow up; m: month. (B) Cryocrit C4 and RF kinetics during the combined therapy (RTX+Peg-IFN+RBV+BOC). The time points considered are (1) before rituximab (June 2011), (2) two months after rituximab cycle (September 2011; triple therapy baseline), and (3) end of the follow-up after boceprevir-based antiviral therapy (November 2013). After RTX therapy cryocrit, C4 and RF normalized and remained persistently normal. (●) Cryocrit; (■) rheumatoid factor; (▲) C4. RF: rheumatoid factor; normal values: <10 IU/mL; normal values of C4: from 10 to 40 mg/dL.

The cryocrit scored negative during therapy and follow-up and C4 and RF remained persistently normal (Table 1 and Figure 1).

During treatment, the patient did not experience purpura or arthritis. She developed anemia and neutropenia requiring administration of erythropoietin and granulokines and reduction of RBV (600 mg/daily).

After therapy, the patient was regularly checked up every three months. During every scheduled check-up C4, RF, cryocrit levels as well as liver functional parameters, and viremia were evaluated (data not shown).

We observed long-term clinical and virological response: after a 15-month follow-up, the patient no longer had purpura, arthritis, or other baseline symptoms. Persistence of mild xerostomia and xerophthalmia was occasionally treated with symptomatic drugs (Table 1).

## 3. Review of the Literature

We performed a literature search on PubMed about combined/sequential treatment of MC (Table 2).

In the first pilot study, Saadoun et al. treated 16 consecutive, unselected refractory HCV-MC patients with RTX followed by AT with Peg-IFN and RBV. 15/16 patients showed clinical improvement, with a good profile of safety [22].

A subsequent study of Terrier et al. comprised 32 severe HCV-MCS patients, treated either with RTX monotherapy (12 patients) or with RTX followed by AT (20 patients, 11 already included in the previous pilot study). The authors reported the efficacy of RTX, with a better response in RTX/Peg-IFN+RBV group [23].

In 2010 Dammacco et al. and Saadoun et al. published two studies on the use of RTX and AT with Peg-IFN+RBV according to a combined [24] or sequential [25] scheme.

In the first study, 37 HCV-MCS patients, naïve for IFNs or previous administration of immunosuppressive drugs, were randomized to get a RTX/Peg-IFN+RBV treatment (22 patients) or Peg-IFN+RBV (15 patients, controls) [24]. Patients in the first group obtained a significantly higher rate of complete and sustained response, without increase of adverse events.

In the second study, a prospective, nonrandomised cohort study of 93 patients, Saadoun et al. found that combined therapy reduced the clinical remission time, improved renal response rate, and led to higher rates of cryoglobulin clearance and clonal VH1-69+B cell suppression than Peg-IFN+RBV alone [25].

In a recent letter, Ignatova and coworkers described 18 patients who underwent AT with Peg-IFN+RBV. In six subjects, RTX was administered 1 month before AT in order to treat severe MC. Data of this report confirm that AT increases the relapse-free survival compared with standard immunosuppression or RTX alone and that combined RTX/AT schedule can be useful in moderate/severe MC [26, 27].

## 4. Discussion

Patients' response to AT has been shown to be lower in HCV patients with MC than without, after both double (Peg-IFN+RBV) and BOC-based triple therapy [15, 28]. The reasons for a lower propensity to eradicate HCV are unknown but are possibly related to a stronger involvement of lymphatic cells (especially B-cell) by the infection in MC [1] leading to more consistent viral reservoirs and, when using DAAs, a higher risk of viral mutations.

The usefulness and safety of RTX in MC have been clearly shown in numerous studies, including also patients with advanced liver disease [11, 21, 29–31], even if most patients treated with RTX-monotherapy experienced vasculitis relapse following B-cell recovery [23].

Combined therapy with RTX plus Peg-IFN+RBV, according to a sequential or contemporary scheme, has previously provided an improvement of clinical response and higher cryoglobulin clearance than Peg-IFN+RBV, in the absence of increasing adverse events [23–25, 32]. The pathogenetic bases could be linked to the association of cooperating mechanisms, like the viral eradication and the depletion of the pathological B-cell clones. Furthermore, the B-cell depletion could favor the elimination of a viral reservoir. On the other hand, the clinical improvement obtained with RTX treatment could make eligible to AT patients previously not eligible.

Interestingly, in our previous study on 35 HCV chronically infected subjects, the only MCS patient who persistently responded to treatment was the one excluded from the comparative analysis owing to a recent treatment with RTX [15].

In the same study we showed a sudden decrease in cryocrit together with the rapid decrease of viremia due to the introduction of BOC, stressing the role exerted by viral replication in MC pathogenesis [15].

In conclusion, we are reporting here the first description of a safe and highly efficacious combination of RTX and BOC-based AT in a patient with cryoglobulinemic vasculitis. The persistent response was both virological and clinical. Our previous observation of a low rate of virological response in MCS patients may increase the interest of this report which seems to agree with the hypothesis of a key role played by B cells as HCV reservoirs. Such an observation appears to confirm the positive effect of a combined therapeutic approach also when using DAA-dependent AT.

These results were, however, obtained in a single case and further studies based on significant numbers of patients are needed in order to confirm our observations in the specific setting of patients with HCV-related MC.

## Figures and Tables

**Table 1:** Main clinical and laboratory data before rituximab (RTX-baseline), 2 months after rituximab cycle (BOC-baseline), and at the end of follow-up after boceprevir-based antiviral therapy (post-BOC).

	RTX-baseline (June 2011)	BOC-baseline (September 2011)	Post-BOC (November 2013)
Gender	Female		
Age (years)	63		
IL28b rs12979860	T/T		
Stiffness (kPa)	14.5	12	11.7
HCV titer (IU × 10^6^/mL)	0.98	1.39	NEG
ALT (IU/L)^†^	44	37	19
MC manifestations			
Clinical			
Purpura	++	−	−
Arthralgia	++	+	−
Weakness	++	−	−
Peripheral neuropathy	−	−	−
Raynaud syndrome	−	−	−
Nephritis	−	−	−
Sicca syndrome	++	+	+
Laboratory			
Cryocrit (%)	5	0	0
RF (IU/mL)^§^	24	<10	<10
C4 (mg/dL)^‡^	9	16	25

RTX: rituximab; BOC: boceprevir; MC: mixed cryoglobulinemia; IL28B: polymorphism of interleukin-28B; stiffness: liver stiffness as evaluated by transient elastography; kPa: kilopascal; IU: international units; ALT: alanine aminotransferase; RF: rheumatoid factor. ^†^Normal values: <40 IU/L; ^§^normal values: <10 IU/mL; ^‡^normal values: from 10 to 40 mg/dL.

**Table 2:** Combined treatment with antiviral therapy and rituximab in MC patients: review of the literature.

Author	Patients	Main indication	Regimen	Therapy outcome (CR/PR/NR)	AEs
Saadoun et al., 2008 [22]	16	Refractory MC	ST (RTX 375 mg/sqm × 4 Peg IFN*α*2b^+^ + RBV^++^ after a month)	(10/6/1)	2°
Terrier et al., 2009 [23]	20	Severe MC	ST (RTX 375 mg/sqm × 4 or 1 gr × 2 Peg IFN*α*2b^+^ + RBV^++^ after a month)	(16/3/1)	4°°
Dammacco et al., 2010 [24]	22	MC (naïve patients)	CT (RTX 375 mg/sqm × 4 weekly + two 5 monthly inf. + Peg IFN*α*2b^+^ or *α*2a^+++^ + RBV^++^)	(12/5/5)	3°°°
Saadoun et al., 2010 [25]	38	MC (unselected)	ST (RTX 375 mg/sqm × 4 or 1 gr × 2 Peg IFN*α*2b^+^ or *α*2a^+++^ + RBV^++^ after a month)	(28/9/1)	5^°°+^
Ignatova et al., 2014 [26]	6	Severe MC	ST (RTX n.d. dose Peg IFN*α* + RBV^++^ after a month)	(n.d./n.d./n.d.)	n.d.

MC: mixed cryoglobulinemia; ST: sequential treatment; CT: combined treatment; CR: complete response, PR: partial response, NR: no response; AEs: adverse events (requiring antiviral treatment interruption); n.d.: not determined.

^+^1.5 mcg/kg weekly; ^++^weight-based; ^+++^180 mcg weekly.

°One worsening of peripheral neuropathy case and 1 flare of psoriasis case.

°°Two hematologic toxicity cases; 1 flare of psoriasis case; 1 hepatocarcinoma case.

°°°One severe anemia case; 2 grade 4 neutropenia cases.

^°°+^Two hematologic toxicity cases; 1 depression case; 1 flare of psoriasis cases; 1 neuropathy case.
